# Sialylation by β-galactoside α-2,6-sialyltransferase and N-glycans regulate cell adhesion and invasion in human anaplastic large cell lymphoma

**DOI:** 10.3892/ijo.2015.2818

**Published:** 2015-01-07

**Authors:** OSAMU SUZUKI, MASAFUMI ABE, YUKO HASHIMOTO

**Affiliations:** Department of Diagnostic Pathology, School of Medicine, Fukushima Medical University, Fukushima, Japan

**Keywords:** sialic acid, glycans, β-galactoside α-2, 6-sialyltransferase, cell adhesion, invasion, galectin-8, Rho, PI3K, MAPK

## Abstract

The interaction between cell surface glycans and extracellular matrix (ECM) including galectins is known to be closely associated with tumor cell adhesion, invasion and metastasis. We analyzed the roles of cell surface sialylation or glycosylation in galectin or ECM-mediated cell adhesion and invasion of human malignant lymphoma cells. Neuraminidase from *Arthrobacter ureafaciens* (AU) treatment resulted in reduction of cell adhesion to galectin-8 in human anaplastic large cell lymphoma (H-ALCL) which was established in our laboratory. The knockdown of β-galactoside α-2,6-sialyltransferase (ST6Gal1) by siRNA showed inhibition of ST6Gal1 expression in the cytoplasm of H-ALCL cells on immunohistochemical findings, and showed dramatic enhancement of cell adhesion to galectin-8. On the other hand, α-2,3-specific neuraminidase treatment resulted in moderate enhancement of cell adhesion to galectin-8. We performed chemically artificial modification of cell surface O-glycans by treatment of benzyl 2-acetamido-2-deoxy-α-D-galactopyranoside (Bz-α-GalNAc) in H-ALCL. Cell adhesion to galectin-8 was enhanced by treatment of Bz-α-GalNAc suggesting that inhibition of elongation of O-glycans may enhance cell adhesion to galectin-8 in H-ALCL cells. On the other hand inhibition of elongation of N-glycosylation by tunicamycin (TM) resulted in inhibition of *Phaseolus vulgaris-L* (L-PHA) lectin-binding activity and inhibited cell adhesion to galectin-8,laminin and fibronectin. Neuraminidase treatment enhanced cell adhesion to laminin, and knockdown of ST6Gal1 resulted in enhancement of cell adhesion to laminin, but not to fibronectin, collagen type 1 and 4. Galectin-8 pre-treatment dramatically enhanced cell adhesion to laminin and neuraminidase treatment also enhanced cell adhesion to laminin in combination with galectin-8. Rho inhibitor, C3-transferase pre-treatment resulted in inhibition of cell invasion to galectin-8. Phosphatidylinositol 3-phosphate kinase (PI3K) inhibitor, wortmannin inhibits the cell invasive capacity to galectin-8. Neuraminidase treatment induces growth inhibition of lymphoma cells by galectin-8.

## Introduction

ST6Gal1 is known to be a regulator for interaction between galectin-1 and N-glycans which possess α-2,6-sialic acid (1) and α-2,6-sialylation plays an important role in many biological processes (2–4). Galectin-8 plays a regulatory role in cell adhesion as an extracellular matrix (ECM) (5–7). We demonstrated that cell surface glycosylation appeared to regulate cell adhesive or invasive properties to galectin-1 in human anaplastic large cell lymphoma (H-ALCL) cell line (1). Cell surface sialic acid or N-glycans regulate adhesive or invasive properties to galectin-1 and linkage of sialic acid is essential to cell adhesive capacity to galectin-1. Furthermore, cell adhesion or invasion through galectin-1 is regulated by phosphatidylinositol 3-phosphate kinase (PI3K), mitogen-activated protein kinase (MAPK), Rho and cytoskeleton (1). Previously, galectin-8 was reported to promote cell movement which is regulated by cytoskeleton (8). Therefore, galectins are needed for cell movement in extracellular space. But biological roles of galectin-8 in human malignant lymphoma are not yet fully understood. In this report we discuss the fundamental roles of galectin-8 in human malignant lymphoma.

## Materials and methods

### Cell line

H-ALCL cell line was established in our laboratory. The cell line derived from a patient has been approved for use by the Bioethics Committee in Fukushima Medical University (Fukushima, Japan). The H-ALCL cells were grown in the culture medium of RPMI-1640 containing 15% fetal calf serum in 5% CO_2_ at 37°C. The H-ALCL cell line expresses the galectin-1 receptors, CD45RA [leukocyte common antigen (LCA), no. 422071; Nichirei Corp., Tokyo, Japan] and CD45RO (UCHL-1, N1520; Dako Japan Co., Ltd., Kyoto, Japan) on the flow cytometric analysis (FACSCalibur; Becton-Dickinson, Tokyo, Japan) (data not shown).

### Cell surface lectin array analysis

We applied the cell surface lectin array analysis to detect the cell surface glycosylations according to Landemarre *et al* with several modifications (9). The H-ALCL cells were treated with or without neuraminidase from *Arthrobacter ureafaciens* (AU) (no. 10269611001; Roche Diagnostics GmbH, Mannheim, Germany) at 0.2 U/ml, at 37°C for 30 min. Then, the cells were cytospun and cytospin cell preparations were stained by lectins. The *Phaseolus vulgaris-L*(L-PHA) lectin was from L-1801-5, EY Laboratories, Inc. (San Mateo, CA, USA). The 96-well plate was coated by each lectin and air-dried. The neuraminidase-treated or non-treated H-ALCL lymphoma cells (1×10^6^/2 ml) were applied to each well (100 μl/well) and incubated at 37°C for 60 min. After aspiration of the medium, PBS was added to each well and then aspirated to remove non-adhered cells. Then 100 μl of 3.7% formaldehyde was added to each well to fix the adhesive cells at RT for 40 min. After aspiration of formaldehyde, 100 μl of 0.1% crystal violet was added to each well and the plates were incubated at RT for 40 min. After washing twice, 100 μl of 10% acetic acid was added to each well and the absorbance at 570 nm was determined using an ELISA plate reader (1). In our previous, unpublished data, PNA and HPA lectin (B-2301-2, L-3601-1, respectively; EY Laboratories, Inc.) reactive oligosaccharides were 2,3-sialylated and L-PHA lectin reactive oligosaccharides were 2,6-sialylated. To analyze the effect of O-glycosylation cells were treated with O-glycosylation inhibitor, benzyl 2-acetamido-2-deoxy-α-D-galactopyranoside (Bz-α-GalNAc B5019; Sigma, St. Louis, MO, USA) at a concentration of 2 mM in culture medium for 72 h at 37°C. Then, Bz-α-GalNAc-treated or non-treated H-ALCL lymphoma cells (1×10^6^/2 ml) were applied to each well (100 μl/well) and incubated as described above. In cell surface lectin array desialylation of O-glycans (PNA, HPA lectin reactivitiy, PNA, B-2301-2 and HPA, L-3601-1; EY Laboratories, Inc.) was validated in our recent report (1). To analyze the effect of N-glycosylation cells were treated with N-glycosylation inhibitor, tunicamycin (TM) (T7765; Sigma), at a concentration of 1.6 μg/ml, for 48 h at 37°C. Inhibition of N-glycans by TM was validated by inhibition of cell adhesion to L-PHA lectin (L-1801-5; EY Laboratories, Inc.) on cell surface L-PHA lectin array analysis.

### Cell adhesion assay

Tissue culture plates with 96 wells were coated with human recombinant galectin-8 (ATGP0385; ATGen, Ltd., Gyeonggi, South Korea) (10 μg/well) and matrix proteins (fibronectin 4305-FN, 0.5, 1.0 μg/well, R&D Systems, Minneapolis, MN, USA; laminin L2020, 10 μg/well, collagen 4 C5533, 50 μg/well and collagen 1 C5983, 10 μg/well, all from Sigma) and dried at room temperature. H-ALCL cells with or without neuraminidase (from AU final concentration 0.2 U/ml, at 37°C for 30 min, and recombinant α-2,3-neuraminidase 1.0, 2.0 and 4.0 U/ml, at 37°C for 30 min, from New England BioLabs, Inc., Ipswich, MA, USA) treatment were added to each well and incubated at 37°C for 1 h. After aspiration of the medium, PBS was added to each well and then aspirated to remove non-adhered cells. Then 100 μl of 3.7% formaldehyde was added to each well to fix the adhesive cells at RT for 5 min. After aspiration of formaldehyde, 100 μl of 0.1% crystal violet (038-17792; Wako Pure Chemical Industries, Ltd., Osaka, Japan) was added to each well and the plates were incubated at RT for 5 min. After washing with PBS, 100 μl of 10% acetic acid was added to each well and the absorbance at 570 nm was determined using an ELISA plate reader (iMark™ Microplate Reader; Bio-Rad, Hercules, CA, USA) (1). For adhesion assay of ECM, laminin (10 μg/well), fibronectin (1 μg/well), collagen type 1 (10 μg/well) and collagen type 4 (50 μg/well) were coated to wells and air-dried.

### Invasion assay

The invasion assay (haptotaxis) was performed based on the methods previously reported (1) with several modifications. The 24-well culture plate was filled with 600 μl the culture medium RPMI-1640 containing 15% BSA and 15% FCS. The lower surfaces of the membranes of Transwell chamber (Chemotaxicell; Kurabo, Osaka, Japan) with 8 μm pore membrane were coated with 10 μl human recombinant galectin-8 (0.125 μg/μl, ATGP0385; ATGen, Ltd.) and air-dried at RT. Then coated Chemotaxi-cells were inserted into each well. The 100 μl of 2.4×10^6^/ml H-ALCL cells were inserted into each Chemotaxi-cell and incubated at 37°C for 24 h. After incubation, the invaded cells of each well were counted by trypan blue exclusion methods. The cell count was performed using triplicate wells with at least two independent experiments. To analyze the effect of cell surface sialylation, H-ALCL cells were treated with neuraminidase from AU (final concentration 0.2 U/ml) at 37°C for 30 min. For analysis of PI3K inhibitor, wortmannin (681675) and MAPK inhibitor, PD98059 (513000) (both from Calbiochem, Darmstadt, Germany) or Rho inhibitor (C3 transferase, CYO-CT04; Funakoshi Co., Ltd., Tokyo, Japan) cells were pre-incubated with wortmannin 1.7 μM or PD98059 25 μM for 1 h, or C3 transferase 2.0 μg/ml, 2 h. Then the cell adhesion or invasion assay was performed. We confirmed the expression of PI3K, MAPK and Rho in the tumor cells of H-ALCL on immunohistochemical staining (data not shown).

### The knock-down of ST6Gal1

In order to analyze the regulatory mechanism of cell surface sialylation by ST6Gal1, siRNA transfection was performed as described previously with several modifications (1). For transfection, INTERFERin (Polyplus Transfection, New York, NY, USA) was used according to the instructions of the manufacturer. For knock-down experiments, siRNA [cat. no. s12842 (called type 42) sense, AGACAGUUUGUACAAUGAAtt and anti-sense, UUCAUUGUACAAACUGUCUtt; or cat. no. s12843 (type 43) sense, ACCACUCAGAUAUCCCAAAtt and antisense, UUUGGGAUAUCUGAGUGGUat; Ambion, Tokyo, Japan] was used. For control experiments, Ambion Silencer™ Select Negative Control no. 1 siRNA (cat. no. 4390843; Ambion) was applied. After 24 h incubation, the immunohistochemical staining was performed by anti-ST6Gal1 antibody (dilution ×100, AF5924; R&D Systems) and knock-down effect was validated by inhibition of ST6Gal1 protein expression in the cytoplasm of H-ALCL cells as shown in our recent reports (1).

### Cell growth inhibitory effects

The H-ALCL cells were grown with or without neuraminidase treatment and then cells were treated with galectin-8 (7.5 μM, ATGP0385; ATGen, Ltd.). After 7 days, the number of viable cells was counted by trypan blue exclusion methods.

## Results

**Figure 1:**
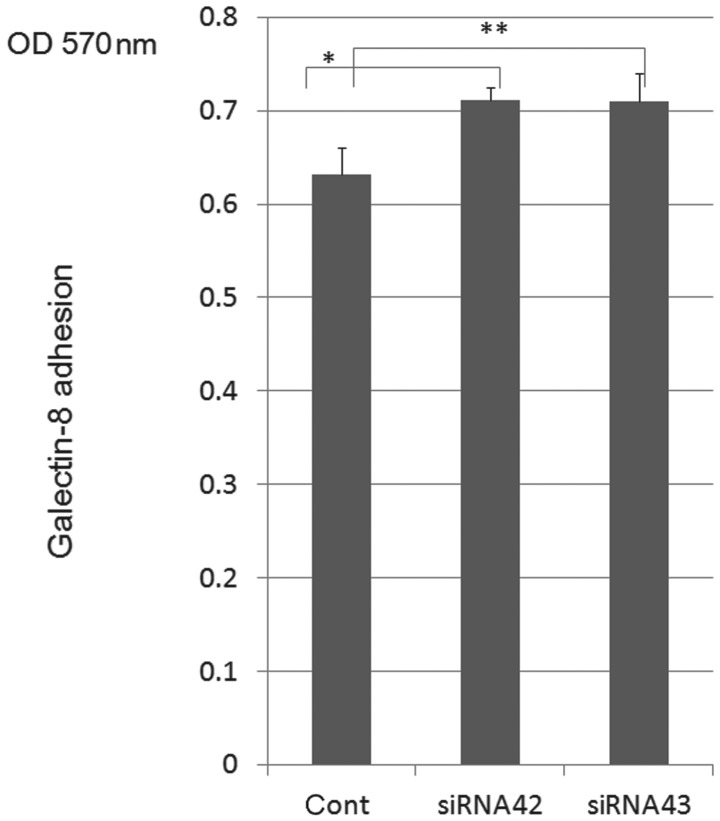
On the knock-down assay for ST6Gal1, knockdown of ST6Gal1 showed enhancement of cell adhesion to galectin-8 (^*^p=0.013, ^**^p=0.016). The data are representative of two independent experiments.

**Figure 2:**
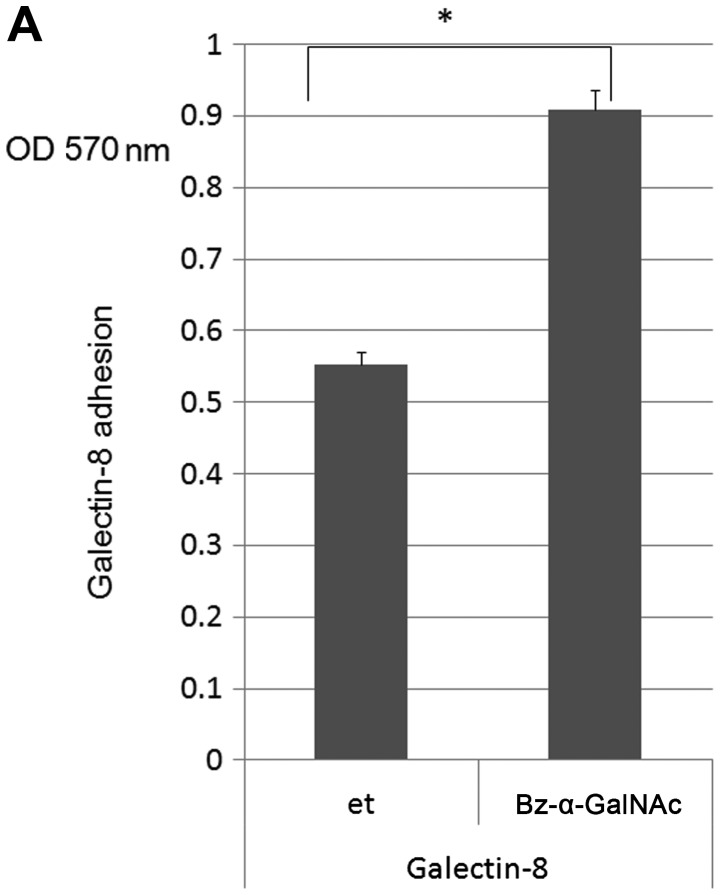

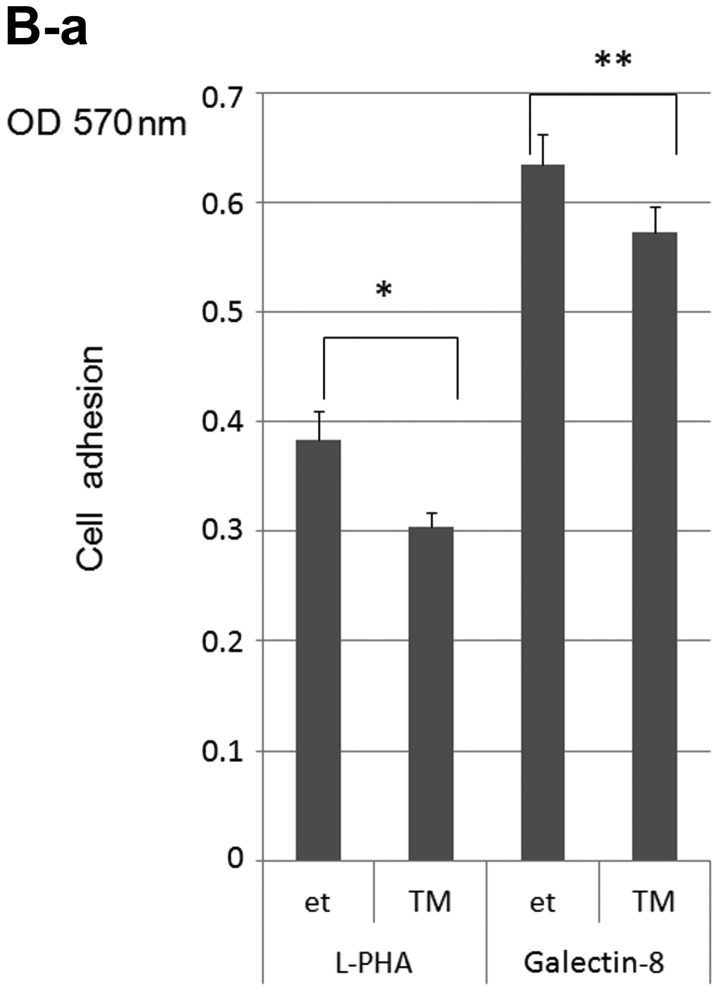

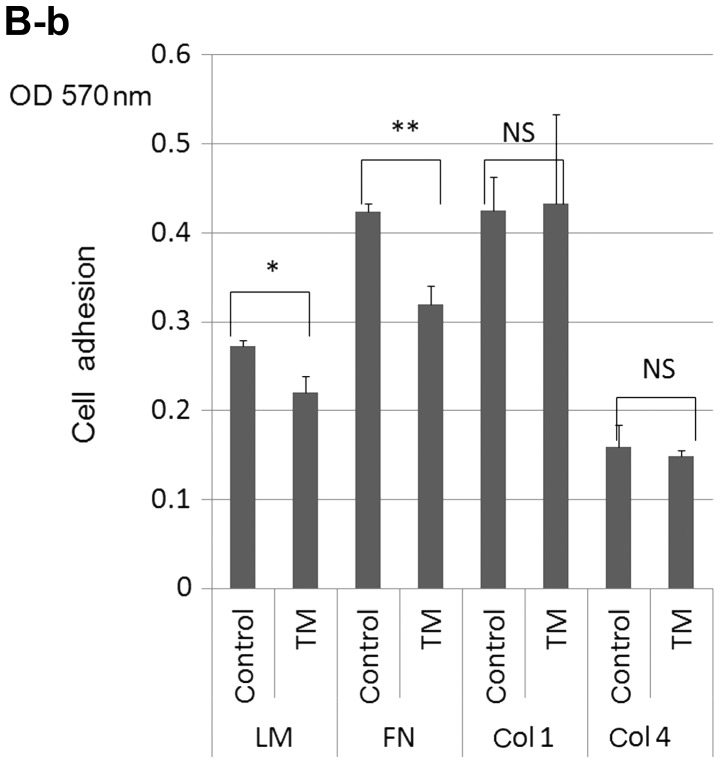

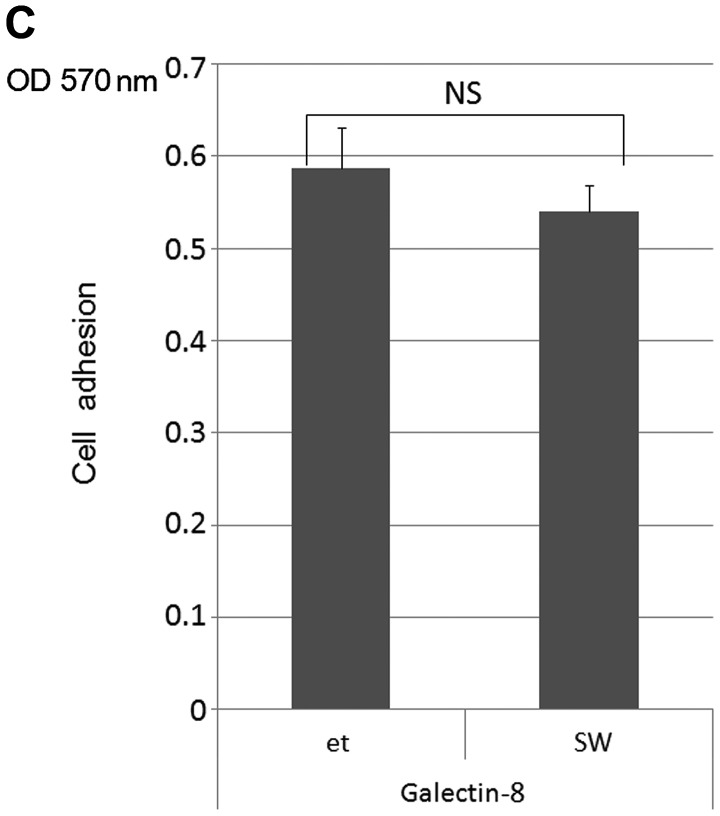
(A) Galectin-8-mediated cell adhesion was dramatically enhanced by treatment of benzyl 2-acetamido-2-deoxy-α-D-galactopyranoside (Bz-α-GalNAc) (^*^p=0.00006). (B) Treatment with tunicamycin (TM) resulted in inhibition of cell binding activity to *Phaseolus vulgaris-L* (L-PHA) and inhibition of cell adhesion to galectin-8, laminin and fibronectin (B-a, ^*^p=0.009, ^**^p=0.02; B-b, ^*^p=0.014, ^**^p=0.002; NS, not significant). (C) N-glycosylation inhibitor, swainsonine did not inhibit cell adhesion to galectin-8 (NS, not significant). The data show representative results from two independent experiments in triplicate.

**Figure 3:**
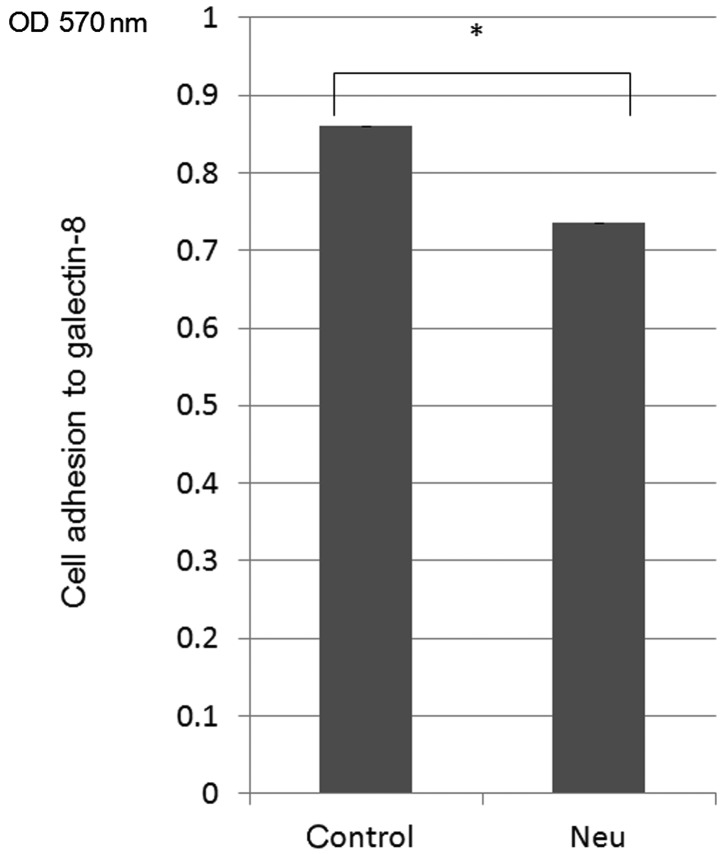
Galectin-8-mediated cell adhesion was inhibited by treatment of neuraminidase (^*^p=0.03; Neu, neuraminidase). The data are representative of two independent experiments.

**Figure 4:**
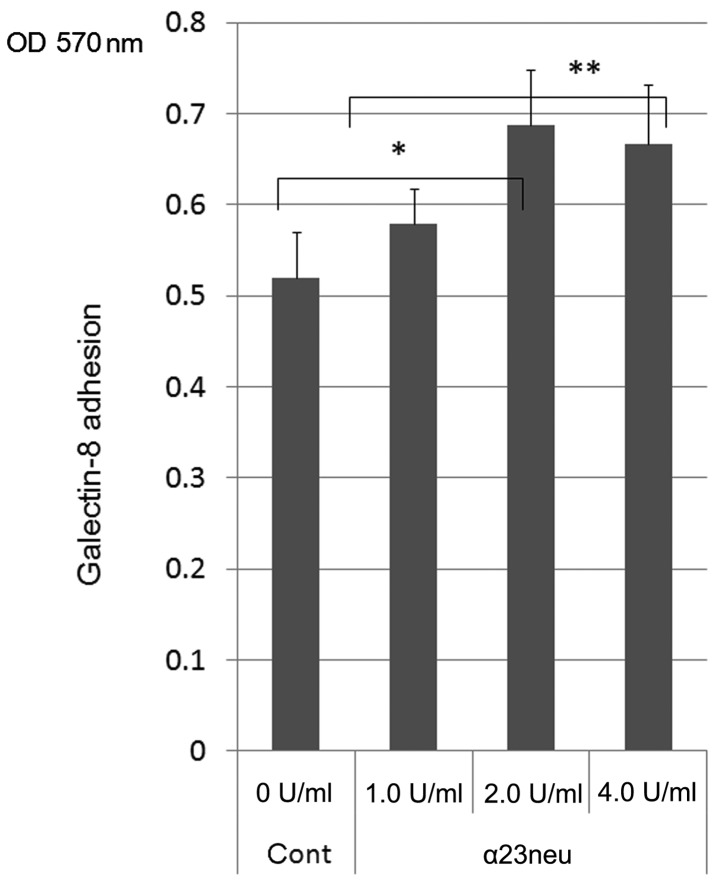
Treatment with α-2,3-neuraminidase resulted in enhancement of cell adhesion to galectin-8 (^*^p=0.01, ^**^p=0.02). The data are representative of two independent experiments.

**Figure 5:**
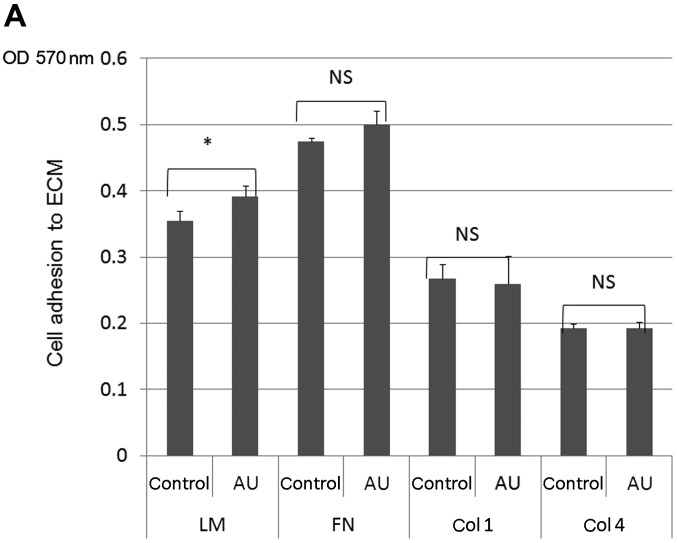

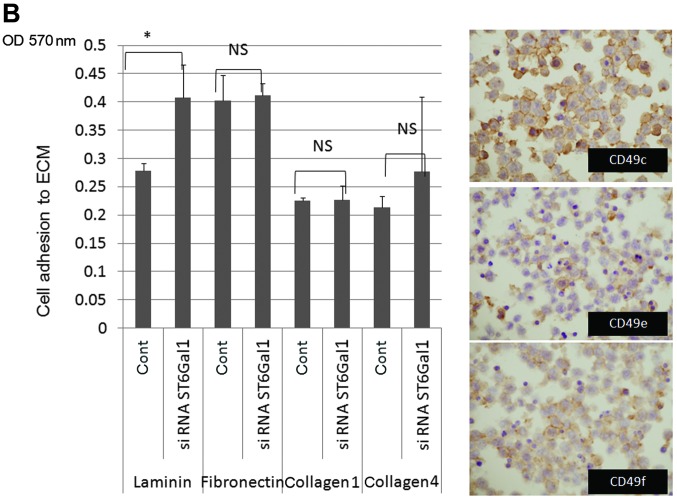
(A) Neuraminidase treatment slightly enhanced cell adhesion to laminin [AU, neuraminidase from *Arthrobacter ureafaciens (AU)* (^*^p=0.049; NS, not significant)]. (B) The knock-down of ST6Gal1 resulted in enhancement of cell adhesion to laminin, but not to fibronectin, collagen type 1 and 4 (^*^p=0.049; NS, not significant). The data are representative of two independent experiments. Human anaplastic large cell lymphoma (H-ALCL) cells expressed integrins; CD49c, e and f in immunohistochemistry.

**Figure 6:**
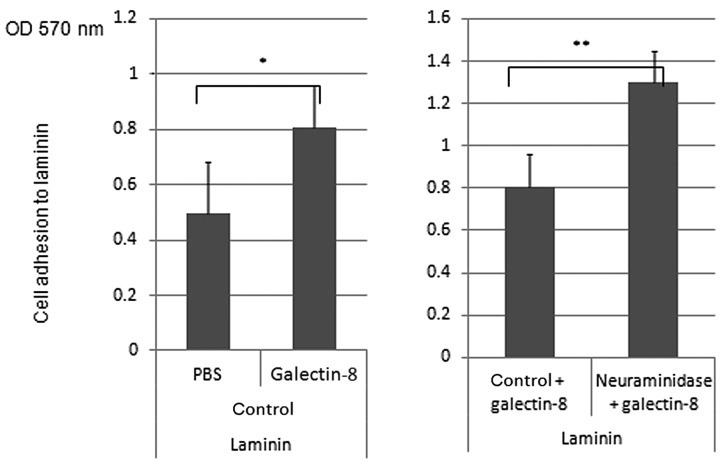
Galectin-8-pre-treatment dramatically enhanced cell adhesion to laminin, and neuraminidase treatment also enhanced cell adhesion to laminin in combination with galectin-8 (^*^p=0.047, ^**^p=0.008).

**Figure 7:**
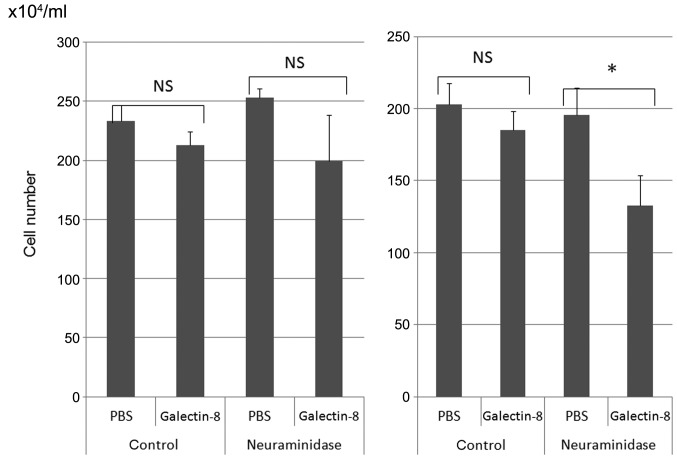
Neuraminidase treatment induces growth inhibition of lymphoma cells by galectin-8 (0.5 μM; right graph, 4 days; left graph, 7 days; ^*^p=0.009; NS, not significant). The data are representative of two independent experiments.

**Figure 8:**
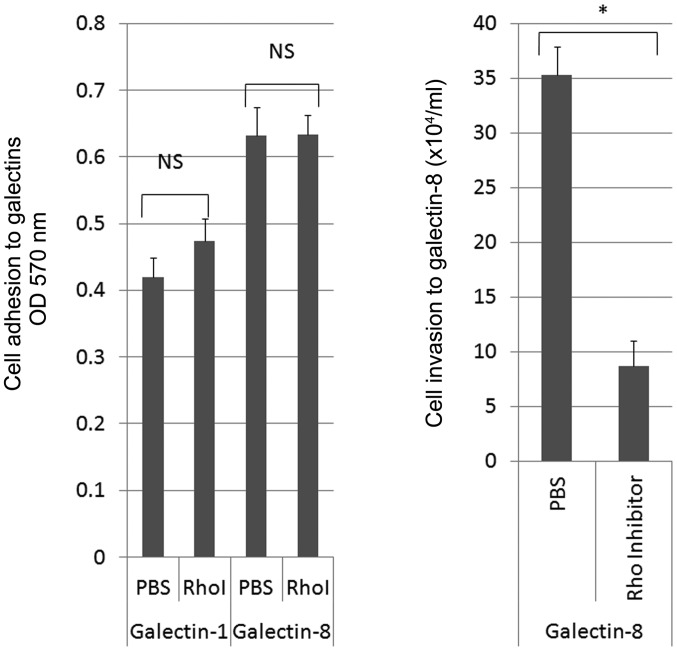
The cell adhesion to galectin-8 was not altered by treatment of the Rho inhibitor (RhoI) C3-transferase (NS, not significant). Pre-treatment of the Rho inhibitor resulted in dramatic inhibition of cell invasion to galectin-8 (^*^p=0.0009). The data are representative of two independent experiments.

**Figure 9:**
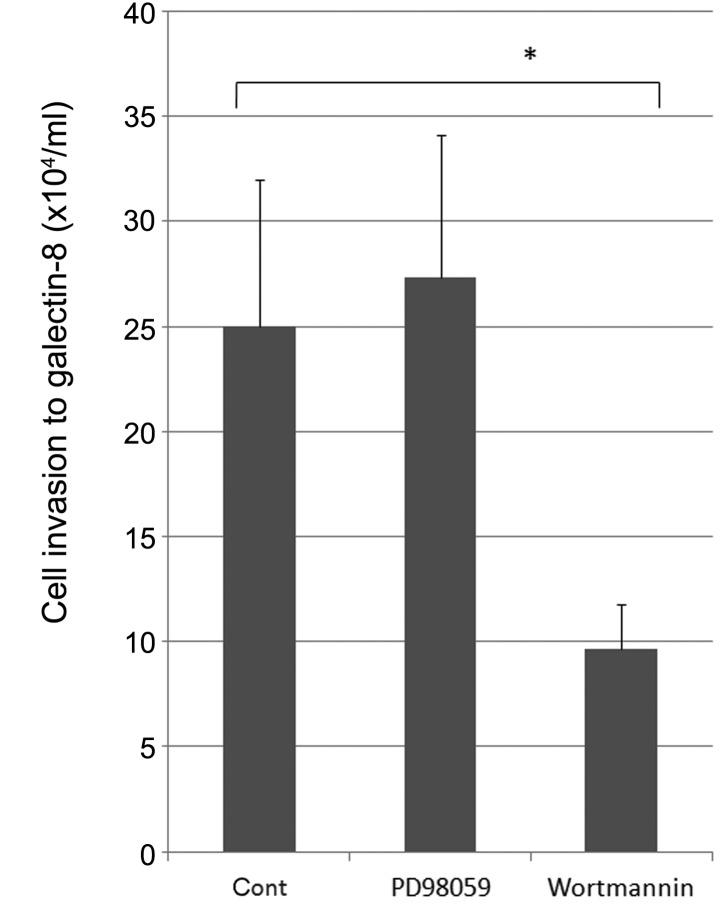
The presence of phosphatidylinositol 3-phosphate kinase (PI3K) inhibitor, wortmannin resulted in inhibition of cell invasion to galectin-8 (^*^p=0.02). The data are representative of two independent experiments.

On the knock-down assay for ST6Gal1, ST6Gal decreased ST6Gal1 protein expression in the cytoplasm of H-ALCL cells [data shown in (1)] and showed enhancement of cell adhesion to galectin-8 (Fig. 1). Galectin-8-mediated cell adhesion was dramatically enhanced by Bz-α-GalNAc treatment (Fig. 2A). Treatment with TM resulted in inhibition of cell binding activity to L-PHA lectin and inhibition of cell adhesion to galectin-8, laminin and fibronectin (Fig. 2Ba and b). The N-glycosylation inhibitor, swainsonine did not inhibit cell adhesion to galectin-8 (Fig. 2C). Galectin-8-mediated cell adhesion was inhibited by treatment of neuraminidase (Fig. 3). Treatment with α-2,3-neuraminidase resulted in enhancement of cell adhesion to galectin-8 (Fig. 4). Neuraminidase treatment slightly enhanced cell adhesion to laminin (Fig. 5A), and knock-down of ST6Gal1 resulted in enhancement of cell adhesion to laminin, but not to fibronectin, collagen type 1 or 4 (Fig. 5B). Galectin-8 pre-treatment dramatically enhanced cell adhesion to laminin and neuraminidase, treatment also enhanced cell adhesion to laminin in combination with galectin-8 (Fig. 6). Neuraminidase treatment induces growth inhibition of lymphoma cells by galectin-8 (0.5 μM, 7 days, Fig. 7). Cell adhesion to galectin-8 was not altered by treatment of Rho inhibitor, C3-transferase, but Rho inhibitor pre-treatment resulted in dramatic inhibition of cell invasion to galectin-8 (Fig. 8). The presence of PI3K inhibitor, wortmannin resulted in inhibition of cell invasion to galectin-8 (Fig. 9).

## Discussion

Based on our previous study, cell surface sialylation inhibits cell death in human lymphoma (10). Oversialylation of cell surface by uridine diphosphate-N-acetylglucosamine 2-epimerase (UDP-GlcNAc2-epimerase), which is a key enzyme in biosynthesis of sialic acid, protects lymphoma cells and promotes cell growth. Neuraminidase treatment or knockdown of UDP-GlcNAc2-epimerase resulted in reduction of cell surface sialylation and showed enhancement of cell death by ceramide. This phenomenon is closely associated with protection of a membrane permeability death by cell surface-masking effect due to sialylation. In our previous study α-2,6-sialylation of L-PHA reactive oligosaccharides is known to be closely associated with a worse prognosis of the patients in human diffuse large B-cell lymphoma (DLBCL) (11). However, biological roles of α-2,6-sialylation of L-PHA reactive oligosaccharides still remain unclear. α-2,6-sialylation of N-glycans may inhibit cell adhesion to galectin-1-facilitating cell invasiveness based our previous research (12). Analysis using patient cases in human DLBCL α-2,6-sialylation of L-PHA reactive oligosaccharides showed a worse prognosis of the patients (11). The data suggested that α-2,6-sialylation of L-PHA reactive oligosaccharide may possess a significant biological role. From the present data α-2,6-sialylation of N-glycans by ST6Gal1 appeared to modulate cell adhesion to galectin-8. This biological phenomenon may be related to lymphoma cell adhesion and motility *in vivo*, because cell adhesion to ECM is known to be closely associated with cell motility based on biological analysis (13). Furthermore, galectin-8-mediated cell adhesion and invasion may play a significant role in lymphoma metastasis, because cell adhesion or invasion which is regulated by cell surface sialylation is known to be related to metastasis, and inhibition of cell adhesion to ECM by cell surface sialylation is known to facilitate release of tumor cells from primary site resulting in a frequent tumor cell distant metastasis *in vivo* (13). This mechanistic model of modulation of cell adhesion to galectin-1 by ST6Gal1 may provide a new concept in understandings the regulatory mechanisms of lymphoma metastasis. Galectin-1 and -8 deposit to extracellular space and H-ALCL cells migrate through interaction between cell surface glycans and galectin-1 or -8. The balance between galectin-1-mediated and galectin-8-mediated adhesion may be associated with movement of lymphoma cells through ECM.

α-2,6-sialylation of glycans on CD45, which is one of the candidates of galectin-1 receptors, is reported to regulate apoptosis induced by galectin-1 with interaction between galectin-1 and CD45 glycans (14). These data suggested that the galectin-1-mediated apoptosis is inhibited by cell surface α-2,6-sialylation, which is regulated by ST6Gal1. In our previous data cell surface N-glycans on CD45 interact with galectin-1 resulting in induction of cell apoptosis (15). Taken together, sialic acid of N-glycans on CD45 may regulate cell apoptosis on human lymphoma cells. The α-2,6-sialylation inhibited tumor cell apoptosis induced by galectin-1 resulting in a more aggressive behavior of human malignant lymphoma.

In the present study treatment of Bz-α-GalNAc enhanced cell adhesion to galectin-8. The lectin array analysis of Bz-α-GalNAc treatment resulted in enhancement of the reactivity of PNA and VVA lectins suggesting that O-glycans were sialylated (1). Bz-α-GalNAc treatment can desialylate the β-galactose residue, which is recognized by PNA lectin, and is a candidate for galectin-8 receptor. On the other hand GalNAc residue which is recognized by VVA lectin is not a galectin-8 receptor. Thus, the findings are complex as to how treatment of neuraminidase inhibits cell adhesive properties to galectin-8. In the other aspects several reports suggested that sialylated β-galactose residue can be a receptor for galectin-8. The cleavage of sialic acid from this structure by treatment of neuraminidase abrogated the binding capacity of galectin-8 to these glycans.

**Figure 10:**
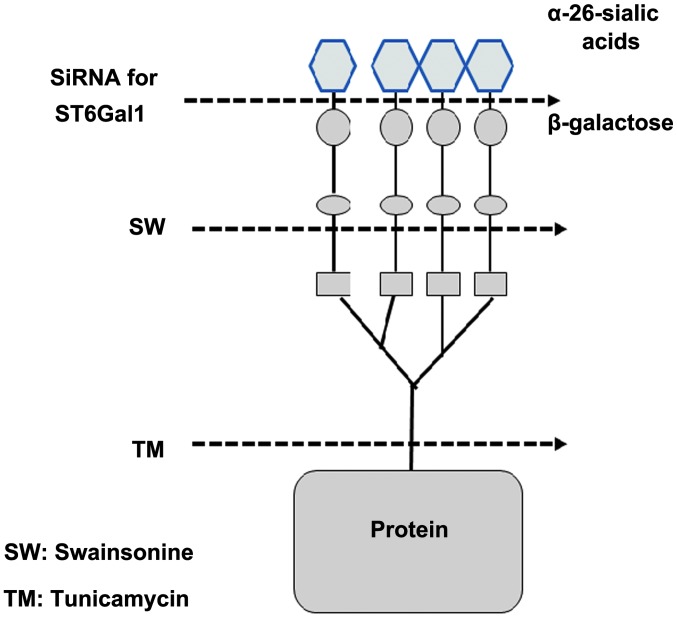
Schematic representation of inhibitory effects of N-glycosylation by glycosylation inhibitors. Treatment of tunicamycin (TM) results in inhibition of elongation of *Phaseolus vulgaris-L* (L-PHA) reactive oligosaccharides.

In the present investigation we clarified the effects of TM in cell adhesion to galectin-8, laminin and fibronectin. Treatment of TM resulted in inhibition of elongation of L-PHA reactive oligosaccharides (Fig. 10) (showing inhibitory effects of cell adhesion to L-PHA lectin) and also inhibition of cell adhesion to galectin-8, laminin and fibronectin. This result suggested that L-PHA reactive oligosaccharides are needed for recognition of galectin-8 on cell adhesion and may regulate cell adhesion to galectin-8 in ALCL. Previously, loss of L-PHA reactive oligosaccharides appeared to be associated with a worse prognosis of the patients in DLBCL (11). Taken together loss of L-PHA reactive oligosaccharides may be related to biological phenomena, such as cell adhesion to galectin-8, laminin and fibronectin, leading to alteration of cell adhesion, invasion and metastasis *in vivo*.

Rho G-protein family is well known to regulate cell motility, especially formation of stress fiber (8). Galectin-1 and -8-mediated cell adhesion is regulated by Rho (8). In the present study treatment of C3-transferase, a Rho inhibitor, resulted in dramatic inhibition of cell invasion to galectin-8. In our speculation galectin-8-mediated cell motility is regulated by Rho and its effector protein, actin cytoskeleton which contributes to formation of stress fiber and contraction of a posterior portion of moving cells. In our recent study PI3K and MAPK are involved in cell motility of ALCL cells (1). The present data indicate that PI3K may be involved in cell invasion to galectin-8 in ALCL. In future investigation Rac1 and Cdc42, which are members of cell motility-regulating proteins will be analyzed in galectin-8-mediated cell invasion in anaplastic large cell lymphoma.

In previous analysis, galectin-1 induced cell death (17–20). Galectin-3-induced apoptosis regulated cell surface sialylation in human DLBCL (21) and galectin-8 induces cell death as reported previously (6,22). In the present study desialylation by neuraminidase treatment induced galectin-8-mediated growth inhibition of lymphoma cells. Based on these data sialic acid may control growth of lymphoma cells by modulation of sensitivity to galectins. In our specultion ALCL cells may escape from galectin-mediated growth inhibition due to masking effects of sialic acid resulting in a more aggressive phenotype and these speculations suggest that oversialylated lymphoma cells are more aggressive and may explain a reason why α-2,6-sialylated L-PHA reactive oligosaccharides are related to a worse prognosis of the patients of DLBCL.
